# New Operation for Obstinate Strabismus

**Published:** 1862-01

**Authors:** E. Andrews

**Affiliations:** Prof. of Surg. in Med. Dep. of Lind University, and Surgeon of Mercy Hospital


### ARTICLE IV.

### NEW OPERATION FOR OBSTINATE STRABISMUS.

By E. ANDREWS, M. D., Prof. of Surg. in Med. Dep. of Lind University, and Surgeon of Mercy Hospital.

Certain cases of strabismus have always been considered incurable; among which are those which result from rupture of the external rectus oculi muscle, and those which are produced by a wound or injury within the internal angle of the lids, fastening the eye by a cicatrix. Yet there occur instances of this sort where the cure is of the utmost importance. Such are those where the sound eye happens, subsequently, to be ruined, while the strabismic one is so turned inward as to be rendered practically useless for supplying its place. In such case the patient is actually in the condition of a blind man, though having one eye perfect in everything but position. Having two such cases on hand, I resolved to try a new operation on one of them. The external rectus muscle had been ruptured, allowing the cornea to turn toward the internal canthus so far as to be quite out of the reach of vision. Some surgeon had made an ineffectual operation on the old plan the only result of which was to form a cicatrix, which glued the eye more firmly into its faulty position.

Having administered ether and chloroform, I commenced, as in ordinary operations for strabismus, by cutting the conjunctiva on the inner side of the eye with the scissors; then, with a blunt hook, I picked up successively every band of cicatrix, tendon or fibrous tissue which interfered with the motion, and cut them off with the scissors until the globe could be freely turned outward, by seizing it with forceps and making traction. The eye was thus liberated from the bond, but as there was no external rectus, there was no voluntary power of keeping it to its place. I proceeded therefore as follows: Pinching up the conjunctiva on the outer side of the globe with the rat-tooth forceps, I cut it perpendicularly with the scissors, two lines from the cornea; commencing at the slit thus made, I dissected off the conjunctiva oculi from the slit outward quite to the lining of the lids at the external angle, denuding the inside and edge of the external canthus at the same time. I then took a silver ligature, bent in the form of a staple, and passed the two ends, first through the strip of conjunctiva remaining next to the cornea, and then through between the eye-ball and external canthus, bringing them out through the skin at two points near the external border of the bony orbit. By drawing upon them, I rolled the eye well out, so as to press the cut edge of the conjunctiva against the denuded canthus, and then fastened the wire by a lead button. In this way the eye was secured firmly in a correct position. Some inflammation followed, which was readily held in check by cold water dressings. On the sixth day the suture was taken out, when the eye was found to maintain its correct position, and the operation proved a complete success.

From the results of this experiment, it is obvious that some cases of strabismus which have hitherto been considered incurable, are capable of being rectified, and that this operation may restore useful vision to a class of patients which had been given over to virtual blindness, by the faulty position of the remaining sound eye.

